# LKB1 tumor suppressor protein regulates actin filament assembly through Rho and its exchange factor Dbl independently of kinase activity

**DOI:** 10.1186/1471-2121-11-77

**Published:** 2010-10-12

**Authors:** Xiaojian Xu, Tatiana Omelchenko, Alan Hall

**Affiliations:** 1 Cell Biology Program, Memorial Sloan-Kettering Cancer Center, 1275 York Avenue, New York, NY 10065, USA

## Abstract

**Background:**

Germline mutations in *LKB1 *result in Peutz-Jeghers Syndrome characterized by intestinal hamartomas and increased incidence of epithelial cancers. *LKB1 *encodes a serine/threonine kinase that plays an important role in regulating energy metabolism through the AMPK/mTOR signaling pathway. In addition, LKB1 is homologous to PAR-4, a polarity protein first described in *C. elegans*, while activation of LKB1 in mammalian epithelial cells induces the polarized assembly of actin filaments.

**Results:**

To explore the mechanism by which LKB1 interacts with the actin cytoskeleton, we introduced LKB1 into HeLa cells that lack endogenous LKB1. This results in activation of the small GTPase Rho and the assembly of linear actin filaments associated with focal adhesions. These effects on the actin cytoskeleton are attenuated by siRNA-mediated depletion of the guanine nucleotide exchange factor Dbl. Co-expression of the LKB1 with the adaptor protein STRAD induces actin filament puncta associated with phospho-ezrin.

**Conclusions:**

This study reveals that LKB1 regulates the actin cytoskeleton through a Dbl/Rho pathway.

## Background

Germline mutations in the gene encoding LKB1, a serine/threonine kinase, results in Peutz-Jeghers Syndrome (PJS), characterized by intestinal hamartomas and increased incidence of epithelial cancers [1]. Inactivating mutations in *LKB1 *have also been found in sporadic human cancers, for example 34% of lung adenocarcinomas and 19% of squamous cell carcinomas [2]. Many cervical cancer cell lines harbor *LKB1 *deletions and expression of LKB1 in the cervical cell line HeLa-S3 (which lack LKB1) is reported to induce a G1 cell-cycle arrest, in agreement with it playing a role as a tumor suppressor [3].

LKB1 regulates several important biochemical pathways, including cell metabolism, cell cycle and cell polarity, but it is not clear which of these are responsible for its tumor suppressor activity. Its ability to regulate metabolic pathways, such as enhanced uptake of glucose and fatty acid oxidation in response to a decrease in cellular ATP levels, is probably the best understood pathway in mammalian cells [4]. In lower organisms, however, its ortholog PAR-4 is best characterized as a polarity determinant. PAR-4 was first identified in *C. elegans *as required for establishing the anterior-posterior axis during cell division of the zygote, while in *D. melanogaster *it regulates polarity establishment in the embryonic epithelium [5-9].

The contribution of LKB1 to cell polarity in mammalian cells has not been extensively explored. The activation of LKB1 in an intestinal epithelial cell line, through over-expression of its adaptor protein STRAD, was reported to induce a polarized morphology in single cells, as visualized by the assembly of an actin-rich brush border on one side of the cell to form an apical-like surface [10]. Further analysis has revealed that the Mst4 kinase and the actin filament binding protein ezrin act downstream of LKB1 in the pathway leading to brush border formation [11]. Since the polarized assembly of actin filaments is a key feature of all epithelial cells, the mechanism by which LKB1 interacts with the actin cytoskeleton is therefore of great interest. Whether the loss of LKB1 seen in epithelial cancers contributes to the tumorigenic process through effects on the actin cytoskeleton remains an interesting possibility.

Members of the Rho GTPase family are important regulators of the actin cytoskeleton and of cell polarity and dysregulated Rho pathways have been linked to the process of tumor progression [12]. Rho and Rac are required for the assembly of cell-cell junctions in a wide variety of epithelial cells, while Cdc42, through its interaction with the Par6/atypical PKC polarity complex, is required for the establishment of apical-basal polarity [13]. So far there have been few reports linking LKB1 to Rho family GTPases and actin. Depletion of LKB1 in migrating non-small cell lung cancer cells, for example, affects Cdc42 activity at the leading edge, though it is not clear if this directly influences actin filament assembly pathways [14]. Intriguingly, Tuberous Sclerosis Protein 1 (TSC1), a protein that acts downstream of LKB1 to control mTORC1 activity, interacts with ezrin and can promote Rho-dependent assembly of actin filaments when expressed in cells [15]. To explore the connection between LKB1 and actin filament assembly, we have introduced an LKB1 expression construct into HeLa-S3 cells, a cervical cancer cell line that lacks endogenous LKB1. We have found this promotes actin fiber formation, through activation Rho *via *the exchange factor Dbl.

## Results

### LKB1 expression induces stress fiber formation in HeLa-S3 cells

**Figure 1:**
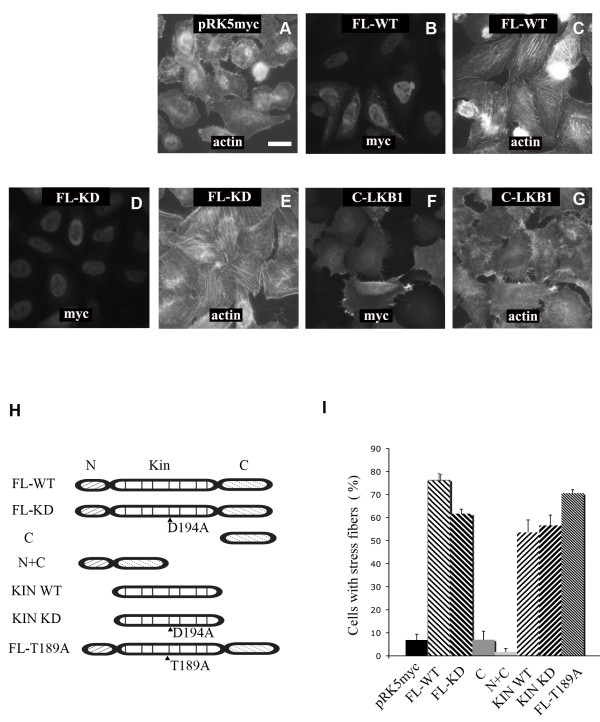
**LKB1 expression induces actin filament assembly in HeLa-S3 cells**. HeLa-S3 cells were transfected with different myc-tagged LKB1 constructs cloned into the pRK5 expression plasmid. Cells were fixed and stained with anti-myc antibody and fluorescent phalloidin to visualize filamentous actin. **A**. Control cells expressing an empty pRK5-myc vector displayed mainly cortical actin filaments and a few thin actin filaments across the cell bodies. **B-E**. Expression of wild type (B and C), or kinase dead (D194A) myc-tagged LKB1 (D and E) led to actin filament formation in HeLa-S3 cells. Both wild type and kinase dead LKB1 were found in the nucleus and in the cytoplasm. **F, G**. LKB1 C-terminus does not induce actin filaments. **H**. LKB1 domain structure and constructs used in the paper. Critical amino acids are indicated. **I**. Quantification of LKB1 effect on actin filament (stress fiber) formation. Expressing cells were scored for stress fiber formation (actin filament bundles across cell bodies). Scale bar = 20 μm.

**Figure 2:**
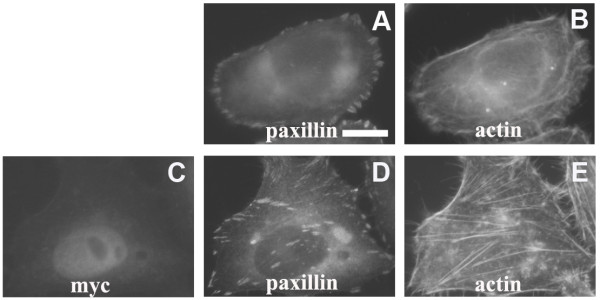
**LKB1 promotes focal adhesion formation in HeLa-S3 cells**. **A.B**. HeLa-S3 control cells stained with paxillin reveal small, peripheral focal complexes, which co-localize with cortical actin. **C-E**. Expression of myc-tagged LKB1 induces elongated focal adhesions throughout the cell body that decorate the ends of newly formed stress fibers. A fraction of overexpressed LKB1 protein is present at the cell membrane. Scale bar = 20 μm.

HeLa-S3 cells contain undetectable levels of endogenous LKB1[3]. To determine whether LKB1 can affect the organization of actin filaments, HeLa-S3 cells were transiently transfected with an LKB1 expression plasmid. As reported previously, LKB1 localizes in the nucleus and in the cytosol (Fig. 1B). 24 hours after transfection, the diffuse thin actin filaments present in control cells (Fig. 1A) were re-organized to form parallel stress fibers traversing cell bodies (Fig. 1C). Visualization of cells with an anti-paxillin antibody revealed that control cells contain predominantly peripheral focal complexes (Fig. 2A, B). LKB1 expression induced more classical, elongated focal adhesions located in the cell body and associated with the ends of actin filament bundles (Fig. 2C, D, E). A small fraction of LKB1 was found to be associated with the plasma membrane (Fig. 2C).

### LKB1 kinase domain is required for stress fiber and focal adhesion formation, but kinase activity is not

To explore the mechanism by which LKB1 induces stress fibers, constructs encoding a series of deletions or kinase dead mutations (Fig. 1H) were used. Two kinase-dead LKB1 mutant constructs (D194A and T189A) were able to induce stress fibers as well as wild type LKB1 (Fig. 1D, E, I). Mutant constructs lacking the kinase domain, however, lost their ability to form stress fibers (Fig. 1F, G, I). A construct encoding only the kinase domain of LKB1 (KIN WT), or a kinase dead, kinase domain (KIN KD) was sufficient to promote stress fiber formation in HeLa-S3 cells (Fig. 1H, I).

### Stress fiber formation requires RhoA

**Figure 3:**
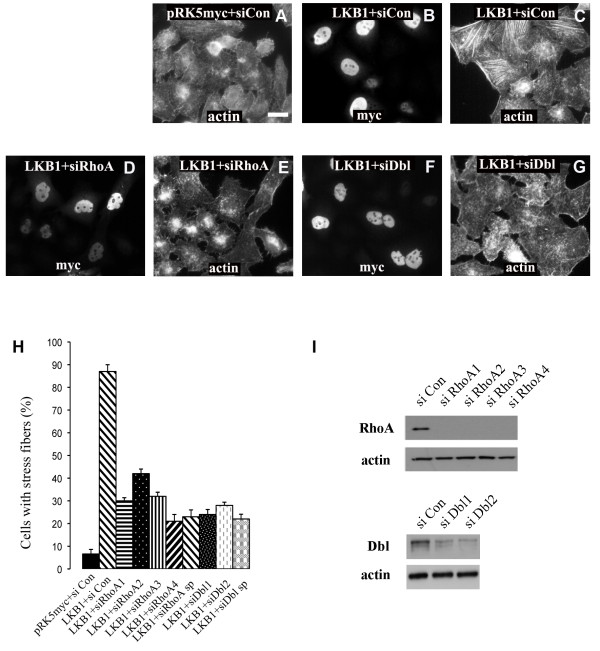
**Stress fiber formation is mediated by RhoA and Dbl**. HeLa-S3 cells were transfected with siRNAs and then re-transfected 2 days later with myc-tagged wild type LKB1. **A**. HeLa-S3 cells transfected with control siRNA followed 2 days later by empty vector. **B, C**. Cells transfected with control siRNA followed 2 days later by a myc-tagged LKB1 construct. **D, E**. Cells transfected with RhoA siRNA SMARTpool followed 2 days later by a myc-tagged LKB1 construct. **F, G**. Cells transfected with Dbl SMARTpool siRNA followed 2 days later by a myc-tagged LKB1 construct. **H**. Quantification of stress fiber formation. Four individual siRNAs (1-4) and the SMARTpool (sp) are shown for RhoA and two individual siRNAs (1,2) and SMARTpool (sp) are shown for Dbl. **I**. Western blot showing effectiveness of individual siRNAs in depleting endogenous RhoA and Dbl proteins. Cell lysates were harvested 3 days after siRNA transfection. Scale bar = 20 μm.

**Figure 4:**
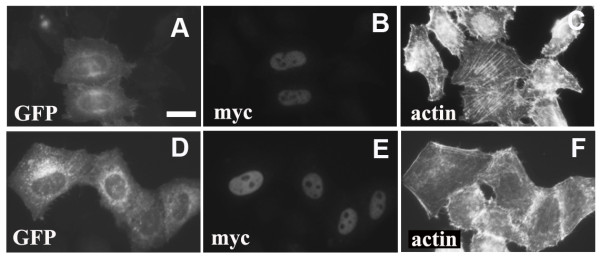
**Dominant negative RhoA inhibits stress fiber formation**. **A-C**. Cells were co-transfected with GFP-DN-Cdc42 (N17Cdc42) and pRK5-myc-LKB1 constructs. **D-F**. Cells were co-transfected with GFP-DN-RhoA (N19RhoA) and pRK5-myc-LKB1. Cells expressing both GFP and myc were counted and scored for stress fiber formation. Scale bar = 20 μm.

Rho GTPases are important regulators of actin filament assembly and their involvement in LKB1-induced stress fiber formation was examined. HeLa-S3 cells were transfected with siRNA SMARTpools targeting different Rho family members (RhoA, RhoB, RhoC, Rac1, Rac3, and Cdc42) and 2 days later cells were re-transfected with a myc-tagged wild-type LKB1 expression construct. Depletion of RhoA prevented stress fiber formation after LKB1 expression (Fig. 3D, E), but depletion of other Rho GTPases had no effect (data not shown). The effect of RhoA depletion by the siRNA SMARTpool was confirmed using the 4 individual siRNA duplexes (Fig. 3H) all of which efficiently depleted RhoA levels (Fig. 3I). Co-transfection of a dominant negative form of RhoA (GFP-N19RhoA) with LKB1 also blocked stress fiber formation (Fig. 4D-F), while dominant negative Cdc42 (GFP-N17Cdc42) did not (Fig. 4A-C). ROCK (rho kinase) is an important RhoA effector involved in actin stress fiber formation [16]. Cells expressing LKB1 were treated with the ROCK inhibitor Y-27632 (10 μM) for 6 hours and this completely inhibited stress fiber formation as expected (data not shown). These experiments suggest that LKB1 induces actin filament assembly through activation of a RhoA/ROCK pathway and that this is direct and not *via *activation of Cdc42 or Rac.

### LKB1 expression results in activation of RhoA

**Figure 5:**
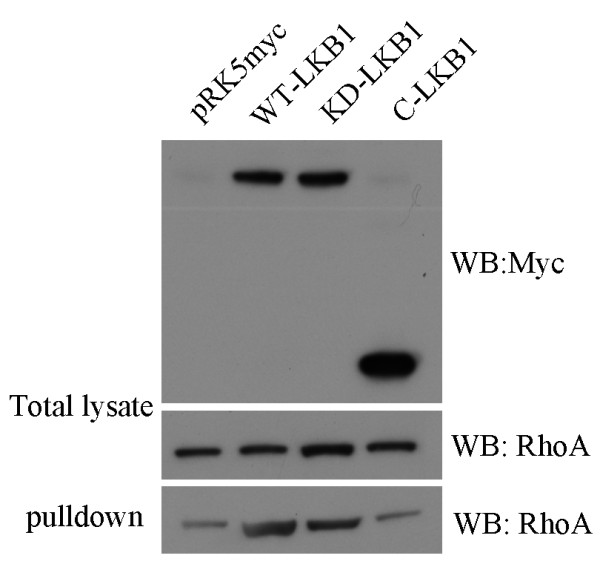
**Expression of LKB1 activates RhoA in HeLa-S3 cells**. Cells were transfected with different LKB1 constructs and 24 hours after transfection, serum-starved for 12 hours. Cells were harvested and activated Rho. GTP levels were determined using a standard pull down assay. Wild type LKB1 and kinase dead LKB1 (D194A) induce a 3-4 fold increase in the levels of active RhoA relative to mock-transfected cells, whereas expression of a C-terminal fragment of LKB1 has no effect.

To determine whether LKB1 expression induces activation (i.e. GTP-loading) of Rho, a Rho. GTP- pulldown assay was performed on HeLa-S3 cell lysates using a GST-rhotekin fusion protein attached to agarose. Expression of either wild type or kinase-dead LKB1 led to a 3-4 fold increase in RhoA.GTP levels, compared to control cells, whereas expression of the C-terminus of LKB1 had no detectable effect (Fig. 5).

### Stress fiber formation requires the exchange factor Dbl

Guanine nucleotide exchange factors (GEFs) catalyze exchange of GDP for GTP on Rho GTPases to activate the conformational switch. The experiments described above raise the possibility that LKB1 activates a GEF specific for Rho [17]. Of the 82 GEFs encoded in the human genome, 22 have been reported to have activity specific for Rho (Additional file 1). HeLa-S3 cells were transfected with siRNA SMARTpools targeting each of these GEFs and two days later, cells were re-transfected with an LKB1 expression construct. Depletion of one GEF, Dbl, efficiently blocked stress fiber formation induced by LKB1 (Fig. 3F, G, H). This effect was confirmed using two Dbl individual siRNA duplexes, both of which depleted endogenous Dbl protein as judged by western blot analysis (Fig. 3H, I).

### The LKB1 kinase domain interacts with DH/PH domain of Dbl

**Figure 6:**
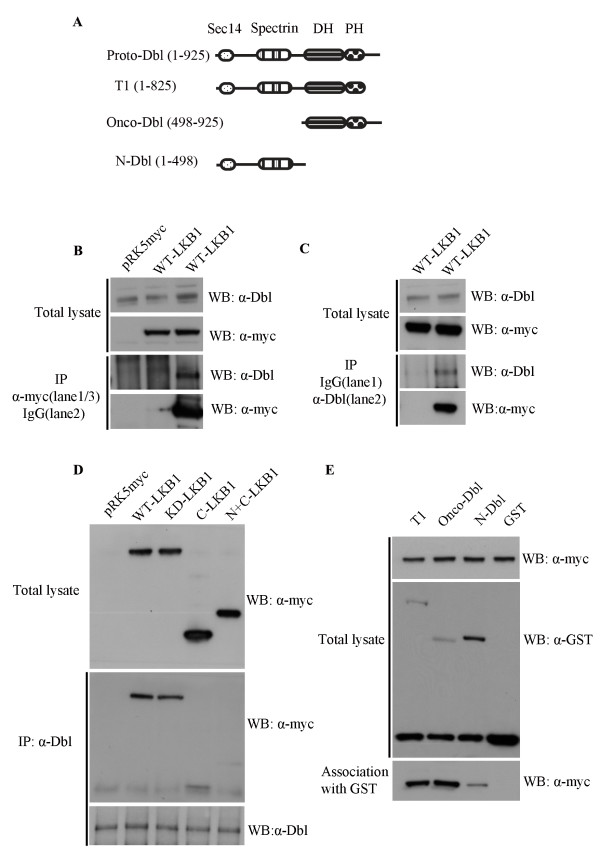
**LKB1 interacts with endogenous Dbl**. **A**. Schematic representation of proto-Dbl and the constructs used in this work. **B**. HeLa-S3 cells transfected with vector (pRK5myc), or pRK5myc-wild type LKB1. LKB1 was immunoprecipitated from cell lysates with anti-myc (lanes 1 and 3), or control IgG (lane 2) antibody and analyzed by western blot analysis. Dbl was detected using a Dbl antibody. Approximately 20% of endogenous Dbl was precipitated with myc antibody. **C**. HeLa-S3 cells transfected with pRK5myc-LKB1. Cell lysates were subjected to immunoprecipitation using either control rabbit IgG (lane 1), or anti-Dbl antibody (lane 2). Upper two panels are total cell lysates blotted with anti-Dbl and anti-myc antibodies. Lower two panels are Dbl and IgG immunoprecipitates blotted with anti-Dbl and anti-myc antibodies. Around 1% of the overexpressed myc-LKB1 was precipitated with endogenous Dbl using anti-Dbl. **D**. HeLa-S3 cells transfected with pRK5myc, or pRK5myc with LKB1 cDNAs. Bottom two panels: Endogenous Dbl immunoprecipitated from cells lysates with anti-Dbl antibody, followed by western blot using either an anti-myc, or anti-Dbl antibody. Top panel: Expression of LKB1 in total cell lysates using anti-myc. **E**. Dbl constructs expressed in HEK293T cells as GST fusion proteins and purified using glutathione agarose. Equal amounts of cell lysate isolated from cells expressing pRK5myc-wild type LKB1 were added to the beads, and bead-associated LKB1 detected on western blot analysis using anti-myc antibody (bottom panel). GST-Dbl and LKB1 were detected with anti-GST and anti-myc antibody, respectively.

The Dbl protein consists of several recognizable domains, including a 200 residue DH domain (Dbl homology) adjacent to a 100 residue PH domain (pleckstrin homology) (Fig. 6A). This DH/PH motif is responsible for catalytic nucleotide exchange activity in Rho family GEFs. To determine whether LKB1 can interact with Dbl, myc-tagged full length LKB1 was expressed in HeLa-S3 cells and immunoprecipitated with an anti-myc antibody or a control IgG antibody. Western blot analysis using an anti-Dbl antibody revealed co-precipitation of endogenous Dbl with LKB1 (Fig. 6B). Similarly, immunoprecipitation of endogenous Dbl with an anti-Dbl antibody led to co-precipitation of myc-tagged LKB1 (Fig. 6C). Next, different myc-tagged-LKB1 constructs were expressed in HeLa-S3 cells and immunoprecipitation reactions carried out with the anti-Dbl antibody. Both wild-type and kinase dead LKB1 (D194A) were found to co-precipitate equally well with endogenous Dbl (Fig. 6D, lanes 2 & 3), but LKB1 constructs lacking the kinase domain did not (Fig. 6D, lanes 4 & 5).

To identify the region of Dbl required for interaction with LKB1, different Dbl constructs (T1, onco-Dbl, N-Dbl, see Fig. 6A) were expressed in HEK293T cells as GST fusion proteins. The proteins were purified from cell lysates using glutathione-agarose beads. Equal amount of beads were then incubated with lysates made from HeLa-S3 cells expressing myc-tagged wild type LKB1. LKB1 was efficiently captured by Dbl constructs containing the DH/PH domains (Fig. 6E, T1 & onco-Dbl), but not by constructs containing only the N-terminal regulatory sequences (Fig. 6E, N-Dbl). We conclude that LKB1 interacts with the DH/PH region of Dbl.

### Co-expression of LKB1 with STRAD leads to the formation of actin puncta

**Figure 7:**
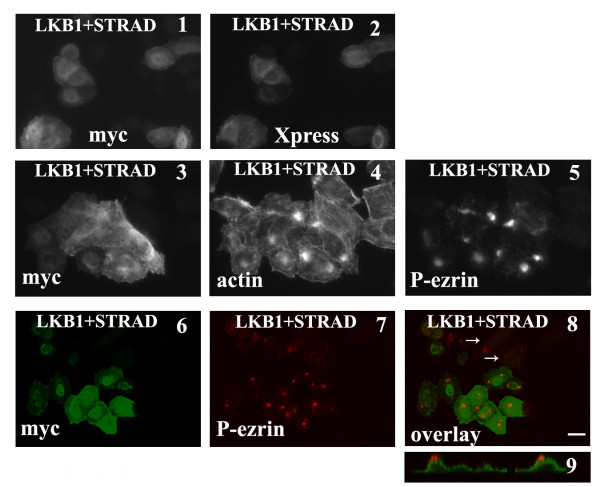
**Co-transfection of LKB1 and STRAD leads to actin puncta associated with phospho-ezrin**. **Panels 1 & 2**: HeLa-S3 cells were transfected with myc-LKB1 and pcDNA-HisMax-STRAD. Cells were stained with anti-myc or anti-Xpress antibodies. LKB1 partially relocated into the cytosol upon STRAD expression. **Panels 3-5**: Cells were transfected with myc-LKB1 and pcDNA-HisMax-STRAD and visualized with anti-myc antibody, anti-phospho-ezrin antibody and phalloidin to visualize actin. Phosphorylated ezrin is associated with actin punctas in transfected cells. **Panels 6-8**: Cells were transfected with myc-LKB1 and pcDNA-HisMax-STRAD and visualized with anti-myc antibody and anti-phospho-ezrin antibody. The basal level of phosphorylated ezrin can be seen in untransfected cells (white arrows, panel 7)**. Panel 9**: Z-section image of cells in panel 7 showing puncta of phosphorylated ezrin present on dorsal surface. Scale bar = 20 μm.

The LKB1 adaptor protein STRAD facilitates nuclear export of LKB1 and is required for full activity [10]. To determine the effect of STRAD overexpression, myc-tagged LKB1 and Xpress-tagged STRAD expression constructs were co-transfected into HeLa-S3 cells. Significantly more LKB1 was found in the cytosol of co-transfected cells than in cells transfected with LKB1 alone (compare Fig. 7, panels 1 & 2 and Fig. 1B). In addition, actin filaments coalesced to form puncta (Fig. 7, panels 3 & 4), which were associated with phosphorylated ezrin (Fig. 7, panels 3-5). Co-expression of STRAD with a D194A kinase dead LKB1 construct produced similar actin puncta (data not shown). Interestingly, the ezrin/actin puncta are localized on the dorsal surface of the transfected cells (Fig. 7, panels 6-8 and z-section in panel 9).

## Discussion

In addition to its well-studied role in the regulation of energy metabolism, the ability of LKB1 to promote cell polarity and actin filament organization is highly conserved across animal species. In Drosophila, *LKB1 *mutations affect apical-basal polarity in a variety of epithelial tissues, including larval wing discs and the retina [5,7]. Further analysis revealed that in some tissues at least, LKB1 acts through 5' adenosine monophosphate-activated protein kinase (AMPK) to phosphorylate myosin light chain (MLC) and LKB1 null mutant show similar disorganized actin as AMPK null mutants. Interestingly, the expression of a phospho-mimetic mutant of MLC was capable of rescuing the polarity defects seen in AMPK-null flies implicating actin:myosin filament assembly as a major player downstream of LKB [5,6]. In agreement with this, the expression of STRAD, an activator of LKB1, in mammalian epithelial cells promotes the assembly of an actin-rich brush border-like structure [10].

Given the importance of actin in LKB1-mediated polarity effects and given the importance of Rho GTPases in actin filament assembly, we have looked for an interaction between LKB1 and Rho GTPases. We introduced LKB1 into HeLa-S3 cells, which lack endogenous LKB1 and found that it promotes the assembly of actin filament bundles or stress fibers. These are characterized by their association with integrin-mediated focal adhesion complexes and by their dependence on Rho kinase activity. LKB1 expression leads to an increase in the activation state of Rho, and this is mediated by the guanine nucleotide exchange factor, Dbl. The overexpression of Dbl has previously been shown to activate Rho and Cdc42, but we found no evidence for Cdc42 activation after LKB1 expression in HeLa-S3 cells. It is also interesting that the kinase activity of LKB1 is not required for the induction of stress fibers, although the kinase domain is. We do not know whether LKB1 interacts directly with Dbl, or whether it is mediated through another protein. Experimentally, constitutive activation of Dbl can be achieved through deletion of the N-terminal 497 residues suggesting that the protein exists in an autoinhibitory state [18]. Relieving autoinhibitory interactions is a common mechanism for activating Rho family GEFs; many have N-terminal sequences encoding recognizable binding sites for regulatory lipids or proteins. However, the mechanism by which Dbl is physiologically activated is not known. It has been suggested that autoinhibition can be relieved through an interaction with ezrin, though it is not clear whether the interaction between ezrin and Dbl is a cause or consequence of Dbl activation [19,20]. Our data suggest that autoinhibition of Dbl may be relieved through binding of LKB1, either directly or indirectly, to the catalytic DH/PH domain, rather than N-terminus.

Interestingly, we found that when LKB1 is co-expressed with STRAD, the actin filaments coalesce into puncta associated with phosphorylated ezrin. These puncta are found on the dorsal surface of cells, somewhat reminiscent of the results reported earlier with STRAD expression in another epithelial cell line [10,11]. Ezrin plays a major role in epithelial cells through its ability to act as a bridge linking actin filaments to the apical plasma membrane [21]. In addition phosphorylated ezrin has been reported to interact with Dbl and activate Rho in T-lymphoma cells [20]. In breast carcinoma cells, ezrin recruits and interacts with Dbl at the plasma membrane, although in this case this results in activation of Cdc42 [22]. We are currently exploring the relationship between ezrin, Dbl and LKB1 in the context of epithelial morphogenesis.

## Conclusions

We have found that LKB1 promotes actin filament assembly through an interaction with the Rho exchange factor, Dbl. We speculate that this may be an important pathway downstream of LKB1 in the establishment of cell polarity in epithelial cells. Disruption of this Rho pathway in cells of patients that lack LKB1 may disrupt the correct organization of actin:myosin filaments and have important consequences for the integrity of epithelial tissues.

## Methods

### Plasmids, siRNAs, antibodies and reagents

Mouse wild type and kinase dead LKB1 (D194A and T189A) in pEF vectors were from Dr. A. Ashworth (Institute of Cancer Research, London) and GST-Dbl plasmids were from Dr. Danny Manor (Case Western Reserve University). pcDNA-HisMax-STRAD plasmid was from Addgene. RhoA and Dbl siRNA SMARTpools (a mixture of four siRNA duplexes) and individual siRNA duplexes were from Dharmacon. The following siRNA oligos were used: RhoA duplex 1: AUGGAAAGCAGGUAGAGUU; RhoA duplex 2: GAACUAUGUGGCAGAUAUC; RhoA duplex 3: GAAAGACAUGCUUGCUCAU; RhoA duplex 4: GAGAUAUGGCAAACAGGAU; Dbl duplex 1: GGAAGAAGUUUAUAUUGUC; Dbl duplex 2: GGUGAUAACCGCAAGUUUG. Primary antibodies used were: RhoA (Santa Cruz-418), β-actin (clone AC-74; Sigma-Aldrich), myc (clone 9E10; Cancer Research UK), GST (Calbiochem), Dbl (Santa Cruz-89); paxillin (Cell signaling-3145); Xpress (Invitrogen 46-0528); P-ezrin (Cell signaling-3144). Secondary antibodies used for immunofluorescence, fluorescent phalloidin and Hoechst 33342 were from Molecular Probes. Secondary antibodies coupled to HRP were from Jackson ImmunoResearch Laboratories.

### Cell culture, transfections and drug treatments

HeLa-S3 and HEK293T cells were maintained in DMEM supplemented with 10% FCS and penicillin/streptomycin and incubated at 37°C and 5% CO_2_. DNA transfections were performed using lipofectamin LTX (Invitrogen) according to the manufacturer's specifications. Briefly, 0.5 × 10^5 ^HeLa-S3 cells were seeded in six-well plates and transfected the next day. For transfections, 5 μl of Oligofectamine™ (Invitrogen) were incubated with 200 μl of Opti-MEM (Invitrogen) for 5 min at room temperature. The mixture was added to 20 nmole siRNA and incubated at room temperature for 15 min before addition to the cells. The medium was replaced the next day. For Rho kinase (ROCK) inhibition, 10 μM Y-27632 (Sigma) was added to cell cultures and incubated for 6 hours.

### Immunofluorescence

For immunofluorescence analysis, cells were fixed in 4% paraformaldehyde for 15 min at room temperature, permeabilized in 0.5% Triton X-100 for 10 min, blocked with 5% BSA in PBS and then stained with antibodies for appropriate epitope tags and fluorescent phalloidin to visualize actin structures. Cells showing actin filament bundles across cell bodies are counted as stress fiber positive, cells showing mainly cortically distributed actin filaments are counted as stress fiber negative. Images were taken with Zeiss microscopy and processed using Axiovision.

### Western blotting and immunoprecipitation experiments

Cells were washed with cold PBS and lysed in standard lysis buffer (50 mM Tris-HCl, pH 8, 150 mM NaCl, 1% NP-40, 0.5% sodium deoxycholate and 0.1% SDS). Cell debris was removed by centrifugation at 13,000 rpm for 10 min at 4°C. SDS sample buffer was added to equal amounts of lysate and proteins were resolved by SDS-PAGE, blotted onto PVDF membranes and then analyzed with the antibodies indicated in the text. For immunoprecipitation experiments, cells were washed with cold PBS and lysed in IP lysis buffer (10 mM Tris, pH 7.4, 140 mM NaCl, 5 mM EDTA, 25 mM NaF, 10 mM sodium pyrophosphate, 1% NP-40, supplemented freshly with 1 mM sodium orthovanadate, 5 mM sodium glycerophosphate, 2 mM PMSF, and one Roche Complete tablet/50 ml). Extracts were cleared by centrifugation at 13,000 rpm for 15 min at 4°C. Protein concentration was measured using the Bradford assay (Bio-Rad Laboratories). For co-immunoprecipitation, 200 μg protein extracts were precleared for 1 h with protein G-Sepharose (Sigma-Aldrich). Extracts were then incubated with 1 μg of antibody overnight, and then with protein G-Sepharose (Sigma) for an additional hour. Beads were washed four times with IP lysis buffer before loading on gels.

### Purification of full length and truncated GST-Dbl fusion protein

48 hours post-transfection with pEBG-GST tagged full length and truncated Dbl constructs, HEK293T cells were lysed by standard IP lysis buffer. Cleared lysates were incubated with glutathione agarose (Sigma) for 1.5 h at 4°C. The beads were washed three times with lysis buffer and GST-Dbl fusion proteins captured on glutathione agarose beads. Cells expressing pRK5-myc-LKB1 were lysed and pre-cleared with GST protein-conjugated glutathione agarose beads for 1 hour. Cell lysates were precipitated and supernatant was collected. GST-Dbl fusion proteins bound to the beads were incubated with the above supernatant at 4°C for another 1.5 h and washed three times. Bead-bound proteins were subjected to PAGE and western blot analysis with an anti-myc antibody.

### GTPase activation assays

1.2 × 10^6 ^HeLa-S3 cells were transfected with 6 μg of DNA expression constructs and serum starved for 12 hours before collection. Cell lysis and pulldown assays were carried out using 20 μg of Rhotekin-RBD as a GST-fusion protein bound to glutathione-sepharose (Sigma). Cell lysates and pulldown samples were run on 12% SDS-PAGE gels and proteins were transferred to PVDF membranes and subjected to western blotting with anti-RhoA antibody.

## List of abbreviations

PJS: Peutz-Jeghers Syndrome; TSC1: tuberous sclerosis protein1; ROCK: rho kinase; AMPK: 5' adenosine monophosphate-activated protein kinase; MRLC: myosin regulatory light chain.

## Authors' contributions

X.X. and A.H. designed experiments; X.X. performed and analyzed the experiments; T.O. performed some of the immunofluorescence experiments; X.X. and A.H. wrote the paper. All the authors have read and approved the manuscript.

## Supplementary Material

Additional file 1 **HeLa-S3 cells were transfected with 22 different GEF siRNA SMART pools. The names and the accession numbers are listed**. Click here for file
