# Supplementary material for: LKB1 tumor suppressor protein regulates actin filament assembly through Rho and its exchange factor Dbl independently of kinase activity
Source: BMC Cell Biol. 2010 Oct 12;11:77. doi: 10.1186/1471-2121-11-77 (PMC2964536; doi:10.1186/1471-2121-11-77)
Supplement: Additional file 1 — HeLa-S3 cells were transfected with 22 different GEF siRNA SMART pools. The names and the accession numbers are listed. [file 1471-2121-11-77-S1.DOC]

**Additional file 1**

**SUPPLEMENTARY TABLE 1**

| Name | Accession # |
| --- | --- |
| GEFT | NM_133483 |
| MCF2 | NM_005369 |
| MCF2L | NM_024979 |
| NGEF | NM_019850 |
| ARHGEF19 | NM_153213 |
| AKAP13 | NM_006738 |
| ECT2 | NM_018098 |
| FARP1 | NM_005766 |
| ABR | NM_001092 |
| BCR | NM_004327 |
| NET1 | NM_005863 |
| ARHGEF12 | NM_015313 |
| ARHGEF2 | NM_004723 |
| ARHGEF18 | NM_015318 |
| ARHGEF1 | NM_004706 |
| ARHGEF17 | NM_014786 |
| ARHGEF11 | NM_014784 |
| VAV1 | NM_005428 |
| VAV2 | NM_003371 |
| VAV3 | NM_006113 |
| RGNEF | XM_371755 |
| FLJ10357 | XM_370737 |
